# Precipitation of Pt, Pd, Rh, and Ru Nanoparticles with Non-Precious Metals from Model and Real Multicomponent Solutions

**DOI:** 10.3390/molecules28135188

**Published:** 2023-07-04

**Authors:** Martyna Rzelewska-Piekut, Zuzanna Wolańczyk, Marek Nowicki, Magdalena Regel-Rosocka

**Affiliations:** 1 Institute of Chemical Technology and Engineering, Faculty of Chemical Technology, Poznan University of Technology, ul. Berdychowo 4, 60-965 Poznan, Poland; zuzanna.g.wiecka@doctorate.put.poznan.pl; 2 Institute of Physics, Faculty of Materials Engineering and Technical Physics, Poznan University of Technology, ul. Piotrowo 3, 60-965 Poznan, Poland; marek.nowicki@put.poznan.pl; 3 Center for Advanced Technology, Adam Mickiewicz University, ul. Uniwersytetu Poznanskiego 10, 61-614 Poznan, Poland

**Keywords:** platinum, palladium, rhodium, ruthenium, platinum-group metals (PGMs), nanoparticles, nanocatalyst, nitrophenol reduction, recycling, spent automotive converters (SAC)

## Abstract

This article presents studies on the precipitation of Pt, Pd, Rh, and Ru nanoparticles (NPs) from model and real multicomponent solutions using sodium borohydride, ascorbic acid, sodium formate, and formic acid as reducing agents and polyvinylpyrrolidone as a stabilizing agent. As was expected, apart from PGMs, non-precious metals were coprecipitated. The influence of the addition of non-precious metal ions into the feed solution on the precipitation yield and catalytic properties of the obtained precipitates was studied. A strong reducing agent, NaBH_4_ precipitates Pt, Pd, Rh, Fe and Cu NPs in most cases with an efficiency greater than 80% from three- and four-component model solutions. The morphology of the PGMs nanoparticles was analyzed via SEM-EDS and TEM. The size of a single nanoparticle of each precipitated metal was not larger than 5 nm. The catalytic properties of the obtained nanomaterials were confirmed via the reaction of the reduction of 4-nitrophenol (NPh) to 4-aminophenol (NAf). Nanocatalysts containing Pt/Pd/Fe NPs obtained from a real solution (produced as a result of the leaching of spent automotive catalysts) showed high catalytic activity (86% NPh conversion after 30 min of reaction at pH 11 with 3 mg of the nanocatalyst).

## 1. Introduction

Nanoparticles (NPs) are a group of materials with at least one dimension not exceeding 100 nm. Depending on size, the material can have completely different properties, e.g., nanomaterials have a larger specific surface area than micromaterials do and therefore have better catalytic properties than micromaterials do [1]. Additionally, the hardness and mechanical strength of pure metal nanoparticles (~10 nm) were found to be up to sevenfold higher than those of metal particles larger than 1 μm [2].

Nanoparticles can be pure metals, metal oxides, metals, silicon compounds, ceramic compounds, organic and biological particles, semiconductors, or polymers [3]. Recently, there have also been many studies on bimetal NPs containing two different metals, e.g., Pt–Cu NPs [4,5,6], Pt–Fe NPs [7,8], Pd–Pt NPs [9,10], and Ag–Fe NPs [11], on metal NPs deposited on supports, e.g., Pt@TiO_2_, Pd@TiO_2_ [12], Pt–Pd@carbon [13], and Pt–Zn@carbon [14], or on nanoliquids, e.g., nanoparticles of CuO, Al_2_O_3_, TiO_2_, or Fe_2_O_3_ dispersed in water or ethylene glycol [3,15,16]. Moreover, atomically monodispersed heterogeneous Pt was proven to be an ideal heterogeneous catalytic material [17]. Nanoliquids are produced by dispersing solid NPs (e.g., metals and metal oxides) in liquids such as water, ethylene glycol, or oils. The large total surface area of the NP results in good thermal transfer properties and the increased stability of the suspension [3].

PGMs (platinum-group metals) are the most expensive metals due to their high demand and scarce natural resources [18,19]. Platinum, palladium, and rhodium form an active layer in automotive converters. The role of the Pt–Pd–Rh layer is to neutralize volatile organic compounds C_x_H_y_, CO and NO_x_ to harmless CO_2_, N_2_ and H_2_O [20]. As the automotive industry is the largest secondary resource of PGMs, it is a crucial issue to recover these valuable metals from spent automotive converters (SAC), and reuse them in new products. Among various pyrometallurgical and hydrometallurgical methods, leaching has often been used to separate PGMs from SAC [21,22,23,24,25,26]. Afterwards, several subsequent steps must be implemented to separate and purify these metals, and form new products, which are cementation [23], ion exchange/adsorption [27,28], and liquid–liquid extraction [25,29]. For example, our team has developed a multistep process for the separation of Pt(IV), Pd(II), Rh(III) and Ru(III) from multicomponent solutions [30,31]. Pt(IV) and Pd(II) can be extracted with Cyphos IL 101 or 104 from acidic solutions obtained after PGM leaching from SAC, and then selectively stripped from the loaded organic phases with 0.1 M thiourea in 0.5 M HCl (for Pd(II)) or 3 M nitric(V) acid (for Pt(IV)). Since the solutions after subsequent steps of the process contain PGMs recovered from SAC in rather small quantities, looking for a way to manage them, we have decided to investigate the leaching and stripping solutions for the precipitation of PGM nanoparticles [10,12].

The aim of this research is to obtain catalytically active PGM NPs (nanocatalysts) from model and real multicomponent solutions. In our previous studies [10], catalytically active PGM NPs were precipitated from one- or two-component model solutions containing PGMs. The scientific novelty of this research is the formation of PGM NPs from real acidic solutions, obtained as a result of the hydrometallurgical processing of spent automotive catalysts, carried out by our team [30]. To the best of our knowledge, this is the first time that acidic leachates have been applied for the formation of PGM NPs. The effect of the addition of non-precious metals (Fe ions, Cu(II), Zn(II) or Mg(II)) into the model feed solutions on the precipitation yield was studied. Afterwards, multicomponent nanocatalysts were formed from the real solutions after metal leaching from SAC. Finally, the catalytic properties of the obtained PGM NPs with the addition of non-precious metals were also investigated.

## 2. Results

### 2.1. One- and Two-Component Model Solutions

In this work, the influence of the addition of non-precious metals to the feed solution was studied on the efficiency of precipitation of PGMs. Fe ions, Cu(II), Zn(II) and Mg(II) were chosen because they are present in real leach solutions (Table A1 in Appendix A).

Initially, the precipitation yield (P) of Fe, Cu, Zn and Mg from one-component model solutions is presented in Table 1.

The precipitation yield of Fe from the one-component solution is 100% using NaBH_4_ or SF as a reducer. Cu can also be precipitated with high efficiency (~80%) using NaBH_4_ or SF. The most efficient reducers for Zn are SF and FA (~90%). AA only precipitates Zn, but the precipitation yield of Zn does not exceed 50%. Regardless of the reducer used, the precipitation efficiency of Mg is poor (<12%). The effect of the type of reducer and the concentration of PVP on the precipitation yield of PGMs from the one-component model solutions (containing Pt, Pd, Rh or Ru) or a mixture of two PGMs (Pt–Pd, Pt–Rh, Pt–Ru, Pd–Rh, Pd–Ru and Rh–Ru) was presented in our previous study [10].

The precipitation yield of PGM with the addition of Fe ions, Cu(II), Zn(II) or Mg(II) from two-component model solutions is presented in Table 2. The molar ratio of PGMs to non-precious metals was 1:1.

On the one hand, the use of a strong reducing agent, NaBH_4_, in the precipitation reaction caused Pt, Pd, Rh, Fe and Cu to precipitate in most cases with an efficiency greater than 90%. On the other hand, AA turned out to be a weak reducing agent with which to precipitate Pd and Rh with high efficiency, which was confirmed in previous studies [10]. When comparing the results of SF and FA, it can be seen that they are similar and that the difference between them is not greater than 10 percentage points in most cases. Slight differences are visible in the PGM–Cu systems; FA turned out to be a weaker reducer than SF was. Due to the position in the table of the redox potentials of Mg(II) and Zn(II) of −2.36 and −0.76 V, their reduction to the zero oxidation state can be difficult. The low metal potential would explain the problem of the efficient precipitation of Mg(II) and Zn(II) (Table 2). In addition, the presence of these metals directly affects the efficiency of reducing PGMs from two-component solutions by lowering the efficiency of reducing all metals [32,33]. The redox potential of PGMs is as follows: Pd^2+^/Pd (0.95 V vs. standard hydrogen electrode (SHE)), Rh^3+^/Rh (0.76 V vs. SHE), Ru^3+^/Ru (0.6 V vs. SHE) and Pt^4+^/Pt (2.28 V vs. SHE). For base metals, it was as follows: Cu^2+^/Cu (0.34 V vs. SHE), Fe^3+^/Fe^2+^ (0.77 V vs. SHE) and Fe^2+^/Fe (−0.44 V vs. SHE) [33,34]. As expected from the reduction potential of PGMs and base metals, only PGMs precipitated from the feed solution, while Mg(II) or Zn(II) remained in solution. According to the potentials, the order of precipitation of PGM ions should be as follows: Pt(IV) > Pd(II) > Rh(III).That for base metals should be Fe(III) > Cu(II) > Fe(II), Zn(II) and Mg(II), which was confirmed by the results obtained in our work.

A strong inorganic reducing agent, NaBH_4_ allows particle production in the metallic form (M) at an alkaline pH (>9) according to the following reaction [35]:

(1) $$4M^{n+}+{BH}_{4}^{-}+8{OH}^{-}\to 4M^{0}+{BO}_{2}^{-}+6H_{2}O$$

The reaction of PGM precipitation with a weak organic reducing agent, i.e., ascorbic acid (AA), proceeds as follows [36]:

(2) $$M^{n+}+nC_{6}H_{8}O_{6}\to M^{0}+nC_{6}H_{6}O_{6}+2nH^{+}$$

As a result of the application of another organic reducer, i.e., formic acid, the reaction of which can be presented as follows [37]:

(3) $$M^{n+}+n{HCOO}^{-}\to M^{0}+{nCO}_{2}↑+nH^{+}$$

**Figure 1:**
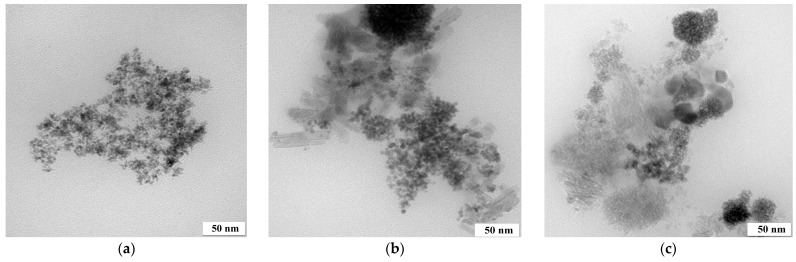
TEM images of (**a**) Pt/Fe NPs, (**b**) Pd/Fe NPs and (**c**) Rh/Fe NPs from the model solution (the molar ratio of PGM:PVP:NaBH_4_ was 1:1:1).

**Figure 2:**
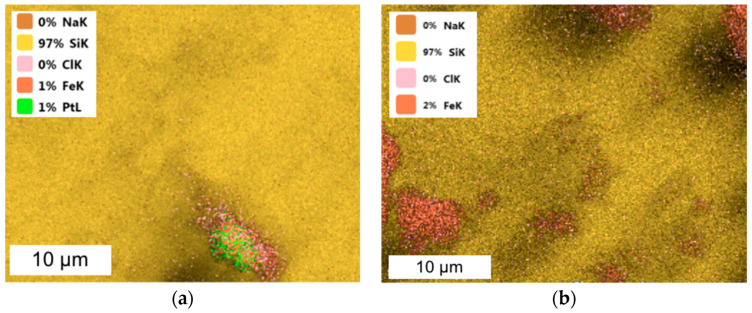
SEM coupled with EDS images of (**a**) Pt/Fe NPs and (**b**) Fe NPs (from model solution; the molar ratio of PGM:PVP:NaBH_4_ was 1:1:1).

The nanocatalysts obtained were characterized via TEM (Figure 1) and SEM-EDS (Figure 2) techniques.

The size of the obtained nanoparticles did not exceed 5 nm. Extensive agglomerate clusters are visible in all images, but Pd and Rh/Fe NPs form larger and more rounded clusters than Pt/Fe NPs do. Similar results for PGM NPs were obtained from one- and two-component solutions and described in our previous article [10]. In the current study, the presence of Fe did not affect the appearance of the obtained PGM NPs. Both PGM and Fe NPs themselves formed agglomerates, which was also confirmed via the TEM images (Figure 1). SEM-EDS was performed for materials containing PGM/Fe or only Fe after reduction with NaBH_4_ (Figure 2).

**Figure A1:**
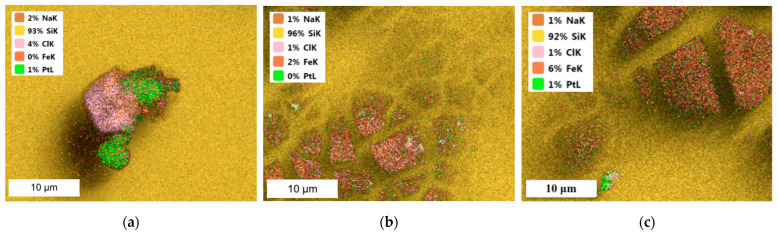
SEM coupled with EDS images of Pt/Fe NPs after reduction by (**a**) AA, (**b**) FA and (**c**) SF from the model solution (the molar ratio of PGM:PVP:reducer was 1:1:1).

**Figure A2:**
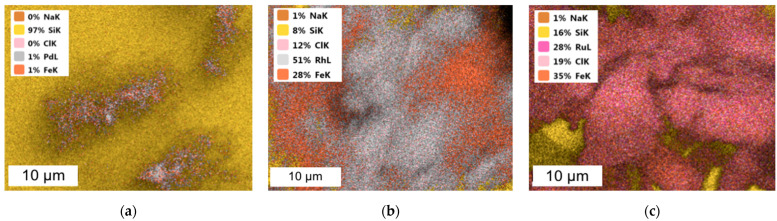
SEM coupled with EDS images of (**a**) Pd/Fe NPs, (**b**) Rh/Fe NPs and (**c**) Ru/Fe NPs from the model solution (the molar ratio of PGM:PVP:NaBH_4_ was 1:1:1).

The SEM-EDS performed confirmed that the metals were reduced to the metallic species and did not form chloride compounds (Figure 2). SEM-EDS images of Pt/Fe nanocatalysts precipitated with AA, FA and SF reducers are shown in Figure A1 in Appendix A. SEM-EDS images for other nanocatalysts are presented in Figure A2 in Appendix A. The absence of Na^+^ and Cl^−^ ions from the EDS images of Pt/Fe NPs and Fe NPs indicates that the synthesized nanocatalyst was well-purified through washing. However, the presence of chlorides was detected in Rh and Ru NPs. The comparison of the results shown in Table 2 indicates that the Ru precipitation from the two-component solution contained Fe ion efficiency by that the NaBH_4_ reducer was below 40% and Ru did not precipitate in the form of metallic NPs or ruthenium oxide. The EDS spectra confirmed the presence of chloride ions in both PGM materials (Figure A2 in Appendix A).

### 2.2. Multicomponent Model Solutions

As in real leachates obtained from various spent materials, PGMs exist in mixtures. The precipitation of Pt, Pd, Ru, and Rh was also carried out from model three- (Pt-Pd-Rh) and four-component (Pt-Pd-Ru-Rh) solutions and the results are presented in Table 3. The molar ratio of Pt:Pd:Rh and Pt:Pd:Ru:Rh was 1:1:1 and 1:1:1:1, respectively.

**Figure 3:**
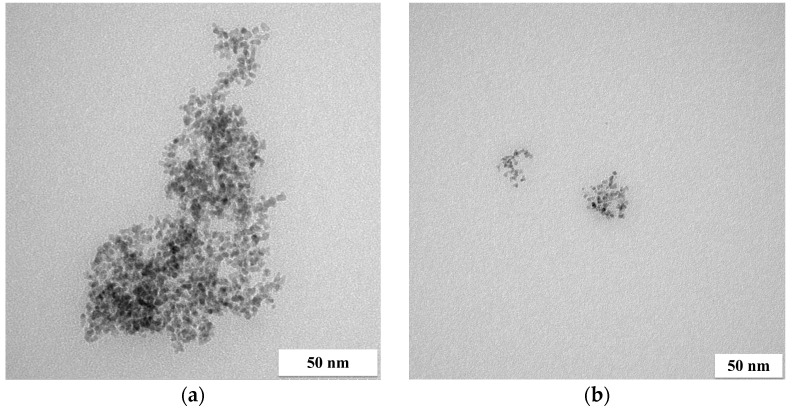
TEM images of four-component NPs from model solution ((**a**) big agglomerate and (**b**) small agglomerate; the molar ratio of PGM:PVP:NaBH_4_ was 1:1:1).

The precipitation of a three-component catalyst with NaBH_4_ or FA was the most effective (above 73%) compared to that with AA and SF. Three-component NPs precipitated with AA contained a small amount of Pd, similar to the case of a two-component nanocatalyst. The order of PGM precipitation from the three-component solution can be written as follows: Pt > Pd > Rh for NaBH_4_, Pd > Rh > Pt for FA and Pt > Rh > Pd for AA. However, this dependence changes when the fourth component is introduced to the solution, in which case the order is as follows: Ru > Pt > Pd > Rh for AA, Pd > Rh > Ru–Pt for NaBH_4_ and Ru > Pd > Pt > Rh for FA and SF. Figure 3 shows the TEM images of the catalysts obtained.

**Figure 4:**
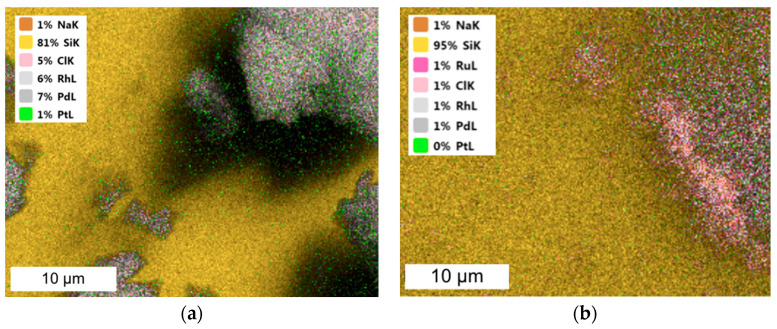
SEM-EDS images of (**a**) three- and (**b**) four-component catalysts precipitated from model solution (the molar ratio of PGM:PVP:NaBH_4_ was 1:1:1).

Some highly developed agglomerates of the four-component NPs were formed, while small groups of metal NPs are visible in other images as well. The size of a single particle did not exceed 5 nm (similarly to the particles obtained from two-component solutions). The materials obtained were characterized using the SEM-EDS technique (Figure 4).

In the case of three- or four-component catalysts, Pd and Rh or Ru agglomerated with one another, while Pt also formed separate clusters, which are visible as the smaller agglomerates present in the TEM images.

### 2.3. Multicomponent Real Solutions

Due to the fact that NaBH_4_ appeared to be the most efficient reducing agent, it was used to precipitate PGMs and other metal ions from multicomponent real solutions. The precipitation of PGMs was carried out from real multicomponent solutions and the results are presented in Table 4.

**Figure 5:**
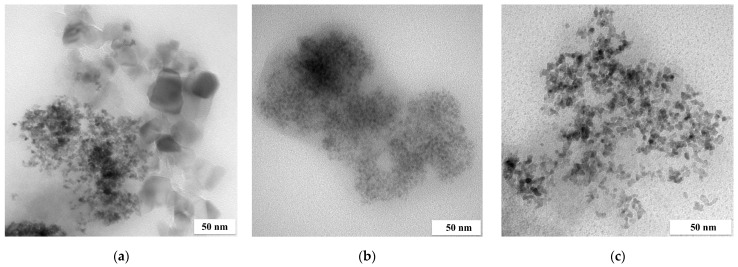
TEM images of (**a**) Pt/Fe NPs from solution R1, (**b**) Pt/Pd/Fe NPs from solution R2 and (**c**) Pt/Pd/Fe NPs from solution R3 (the molar ratio of PGM:PVP:NaBH_4_ was 1:1:2 or 1:1:10).

The concentrations of metal ions in the R1–R3 feed solutions are shown in Table A1 in Appendix A. A 3 M HNO_3_ solution was used as the stripping phase for R1 and R3 and 0.1 M thiourea in 0.5 M HCl were used for R2. The efficiency of the precipitation of PGMs and base metals from the real solutions was almost quantitative. Only the efficiency of Fe precipitation from solution R2 was below 70%. Due to problems during the precipitation of Pd(II) from the R2 solution, a fivefold excess of the reducing agent was applied. This may have been due to the presence of thiourea in the feed solution, the particles of which strongly coordinate PGMs, and, thus, the presence of thiourea affects the reducing capacity of PGMs via NaBH_4_ [38]. TEM images characterizing the formed nanocatalysts are shown in Figure 5.

Additionally, in the case of precipitation from real multicomponent solutions, the size of a single nanoparticle is not larger than 5 nm. The materials obtained from the R1 and R3 solutions look similar, while a kind of shell (visible in all the images obtained is formed) around the Pd NP catalyst was synthesized from the R2 solution. Additionally, it is impossible to see the full outline of the NPs because they look blurred.

### 2.4. Catalytic Reactions

**Figure 6:**
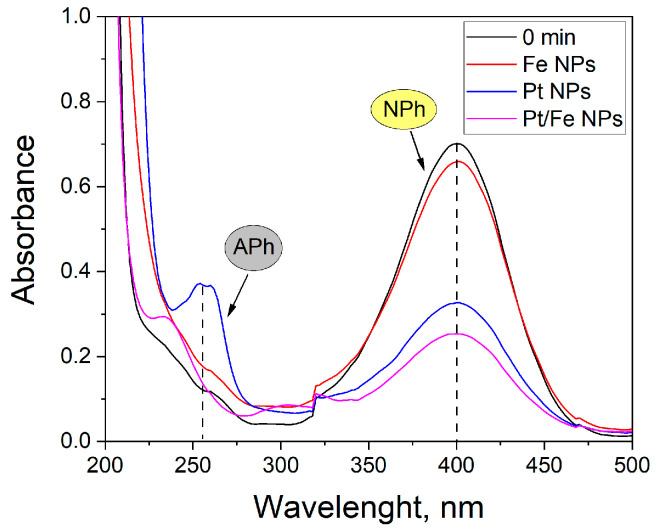
UV-Vis spectra of NPh solutions at pH 11 after 30 min of reduction reaction catalyzed by 1 mg of Fe NPs, Pt NPs and Pt/Fe NPs.

The catalytic activity of the obtained materials was tested via the catalytic reduction of nitrophenol (NPh) to aminophenol (APh) (Table A2 in Appendix A). Due to the change in the reaction mechanism of the NPh reduction with the increasing pH [12], the reactions were carried out at two different pHs, 11 and 14. The highest catalytic activity was observed for Pd/Fe, Rh/Fe and Cu NPs at pH 11. An increase in the reaction pH caused the decrease in catalytic activity of Cu NPs and probably converted copper into its hydroxide, as evidenced by the green color of the solution. Therefore, further research should be mainly focused on Fe addition to newly synthesized PGM NPs. It was observed that the increase in pH to 14, resulted in an improvement in the NPh conversion catalyzed by Pt/Fe NPs and Zn. Figure 6 shows the UV-Vis spectra of the NPh reduction reaction using Pt NPs, Fe NPs and Pt/Fe NPs as a catalyst.

The nanoparticles used for the reduction reaction have catalytic properties. After 30 min of the reaction, the conversion of NPh with the use of one-component catalysts was 5% for Fe NPs, and 52% for Pt NPs. The application of a two-component Pt–Fe catalyst improved substrate conversion by 12 percentage points.

The NPh conversion values with various amounts of PGMs/Fe NPs (1, 2 and 3 mg) are shown in Table A3 in Appendix A. The pH and the amount of the catalyst (Pd and Rh/Fe NPs) had no effect on the increasing conversion of NPh.

**Figure 7:**
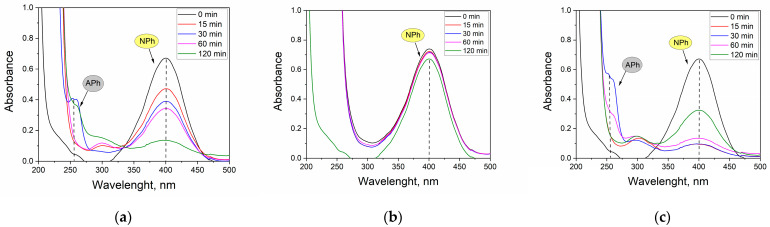
UV-Vis spectra of NPh solutions at pH 11 after 120 min of reduction reaction catalyzed by 3 mg of a catalyst from real solution: (**a**) R1, (**b**) R2 and (**c**) R3.

Finally, nanocatalysts precipitated from real solutions (R1–R3) were used to reduce NPh to APh (Figure 7).

The maximum at λ = 400 nm corresponds to the dissociated form of NPh found under alkaline conditions (pH 11) and that at λ = 313 nm corresponds to the undissociated form of NPh. Already after 15 min of the reaction, a decrease in absorbance for NPh for R1 and R3 could be seen. In both cases, there was also a slight maximum at λ = 300 nm, which is characteristic of APh in its undissociated form. After 30 min of the reaction, a maximum appeared at λ = 260 nm, corresponding to the dissociated form of APh. The conversion of NPh after 30 min was 42.2 and 85.7%, and after 120 min it was 80 and 51.7%, for Pt/Fe NP and Pt/Pd/Fe NP catalysts, respectively (precipitated from R1 or R3 solution). The Pt/Pd/Fe NP catalyst formed from the R2 solution showed no catalytic activity. This may have been due to the presence of thiourea, which may have blocked the active sites of the catalyst. This would explain the casing visible behind the TEM images of R2 (Figure 5b).

## 3. Materials and Methods

### 3.1. Reagents and Solutions

Two-component model solutions (PGM–non-precious metal) were prepared by dissolving in 0.1 M HCl the required amounts of PtCl_4_ (96%), PdCl_2_ (99.9%), RhCl_3_ (99.9%), RuCl_3_ (99.9%), FeCl_3_ (97%), ZnCl_2_ (98%), MgCl_2_ (97%) and CuCl_2_ (97%) (Sigma Aldrich, Schnelldorf, Germany). Polyvinylpyrrolidone (PVP, M_w_ ≈ 55,000, Sigma Aldrich, Schnelldorf, Germany) was used as the stabilizing agent. Sodium borohydride (NaBH_4_) (>98.0%, Sigma Aldrich, Schnelldorf, Germany), ascorbic acid (C_6_H_8_O_6_) (AA, p.a., Chempur, Piekary Śląskie, Poland), sodium formate (HCOONa) (SF, p.a., Sigma Aldrich, Schnelldorf, Germany), and formic acid (HCOOH) (FA, p.a., Sigma Aldrich, Schnelldorf, Germany) were used as the reducers in the study.

The real solutions used in the research as feed solutions were from the hydrometallurgical treatment of spent automotive converters. As the material, two different spent automotive catalysts containing Pt–Rh and Pt–Pd–Rh were used, which were leached in the first stage with oxalic acid in order to purify the material from base metals, and then in the second stage with a mixture of concentrated acids with an oxidant HCl/H_2_SO_4_/H_2_O_2_. The separation and purification procedure was presented in the previous article [30]. The real solutions applied for NP formation (Section 2.3) originated from the stripping step and were used to separate Pd(II) from Pt(IV) and non-precious metals, with 3 M HNO_3_ for R1 and R3 and 0.1 M thiourea in 0.5 M HCl for R2. The concentrations of metal ions in the feed solutions used for precipitation are shown in Table A1.

### 3.2. Synthesis of Nanoparticles

In our previous study [10], the effect of PVP concentration, the type of reducer, and the addition of other PGMs to the feed solution on the precipitation yield of PGMs was studied. The stabilizing agent PVP was added to the PGM precursor solution and mixed for 10 min. Next, the reducer was added drop by drop, and the solution was still mixed. The pH of the solution was then adjusted to pH 7–8 with 1 M Na_2_CO_3_ (p.a., Chempur, Poland, for model solutions) or 30% NaOH (p.a., Chempur, Poland, for real solutions). The molar ratio of the PGM precursor to the stabilizing agent and the reducer for the model solutions (PGM:PVP:reducer) was 1:1:1 and that for the real solutions R1 and R3 was 1:2:1. Due to the lack of color change in the solution and the low precipitation yield (P) for R2, the concentration of the reducing agent was 10 times higher. The precipitation yield (P) was calculated as in the previous studies [10].

### 3.3. Catalytic Reaction

The precipitated NPs were used as catalysts in the reduction reaction of 4-nitrophenol (NPh) to 4-aminophenol (APh) in the presence of NaBH_4_.

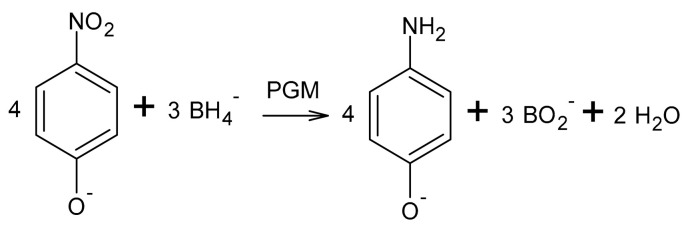
(4)

NaOH was added to the solution to raise the pH to 11 or 14 and keep NPh in an anionic form. Additionally, due to the differences in the mechanism, the reactions were carried out at two different pHs. At a pH above 13, the reaction was dominated by slow hydride reduction. In contrast, at pH 10, the reaction was dominated by rapid hydrogen reduction and exhibited typical surface reaction behavior. Therefore, the use of two different pH values (11 and 14) was performed to investigate the effect of the course of the reaction [39]. The maximum of the NPh in the UV-Vis spectrum in the basic solution is at λ = 400 nm, and that for APh is at λ = 260 nm. The equation for calculating the NPh conversion degree (α_NPh_) was presented in the previous study [10].

### 3.4. Apparatus

The concentrations of metal ions in the solutions before and after precipitation were measured using atomic absorption spectrometry (AAS ContrAA 300, Analytik Jena, Jena, Germany) at the following wavelengths: 266.0, 244.8, 343.5, 349.9, 248.3, 285.2, 213.9 and 324.7 nm for Pt(IV), Pd(II), Rh(III), Ru(III), Fe ions, Mg(II), Zn(II) and Cu(II), respectively.

SEM-EDS (SEM FEI Quanta 250 FEG, ThermoFisher, Hillsboro, OR, USA) and the Hitachi HT7700 transmission electron microscope (Hitachi, Tokyo, Japan) working in high-contrast and high-resolution mode were applied to analyze the structure and morphology of the obtained nanoparticles. The solutions after the reduction of NPh to APh were analyzed via UV-Vis (spectrophotometer, Specord 40, Analytik Jena, Jena, Germany) to confirm the conversion of NPh.

## 4. Conclusions

The research conducted confirms that it is possible to obtain catalytically active nanomaterials from processing spent automotive converters. The most effective reducing agent is NaBH_4_ with a precipitation efficiency greater than 80% for Pt, Pd and Rh NPs from three- and four-component solutions. Due to the high efficiency of PGM precipitation, NaBH_4_ was selected to reduce metal ions from real solutions, and the efficiency of precipitation was almost quantitative. The size of single NPs did not exceed 5 nm in all cases. The presence of Fe in the Pt/Fe NP catalyst improved the NPh conversion (52% conversion of NPh with Pt/Fe NPs) compared to the 64% NPh conversion with Pt NPs. The catalyst containing Pt, Pd and Fe NPs obtained from the real solution had a satisfactory catalytic activity, which was confirmed by the NPh conversion of 86% after 30 min at pH 11 with the use of 3 mg of the catalyst. Thus, this shows that there is no need to purify real leach solutions from ions of non-precious metals before the precipitation of PGM NPs. However, other species (e.g., organics) present in the solution can negatively influence the activity of the NPs formed, for example the blocking of the surface of the precipitated material via the thiourea present in the stripping solution R2.

To the best of our knowledge, there are no literature data on PGM NP precipitation from real acidic leachates. Such a waste-to-product procedure is an important approach from the point of view of sustainability and protection of the environment.

## Figures and Tables

**Table 1:** Precipitation yield of Fe, Cu, Zn and Mg from one-component model solutions using four reducers: AA (ascorbic acid), NaBH_4_, SF (sodium formate), and FA (formic acid) (the ratio of non-precious metal:PVP:reducer was 1:1:1).

Reducer	P, %			
	Fe	Cu	Zn	Mg
AA	0	0.6	46.4	8.1
NaBH_4_	100	80.7	27.2	6.3
SF	100	78.7	87.8	7.8
FA	85.2	69.9	93.2	11.6

**Table 2:** Precipitation yield of Pt, Pd, Ru, Rh, Fe, Cu, Zn and Mg from two-component model solutions (PGM–non precious metal: Fe, Cu, Zn or Mg ions) using four reducers: AA, NaBH_4_, SF and FA (the ratio of PGM:PVP:reducer was 1:1:1).

Reducer	P, %			
	Pt/Fe	Pd/Fe	Ru/Fe	Rh/Fe
AA	81.8/11.6	0/0	40.4/40.5	20.1/55.3
NaBH_4_	88.5/100	100/100	39.7/100	99.7/100
SF	85.4/100	74.8/100	63.7/100	19.8/64.3
FA	82.7/100	93.3/100	67.4/100	15.2/57.9
	**Pt/Cu**	**Pd/Cu**	**Ru/Cu**	**Rh/Cu**
AA	62.4/6.5	8.7/0	67.2/73.1	20.4/78.8
NaBH_4_	97.7/90.4	29.8/37.2	68.4/93.5	50.1/95.3
SF	58.3/91.6	100/98.9	83.5/95.6	38.9/68.2
FA	67.6/89.8	100/27.7	85.0/92.6	43.0/59.5
	**Pt/Zn**	**Pd/Zn**	**Ru/Zn**	**Rh/Zn**
AA	59.0/18.7	1.8/0	64.4/0	30.5/76.9
NaBH_4_	91.6/27.1	100/51.0	63.3/61.1	99.1/57.2
SF	76.1/71.5	100/50.3	81.1/61.4	24.1/29.3
FA	75.3/68.9	100/53.0	79.9/54.0	26.4/6.8
	**Pt/Mg**	**Pd/Mg**	**Ru/Mg**	**Rh/Mg**
AA	70.0/6.4	0/7.1	72.0/1.4	0/0
NaBH_4_	76.5/0.9	62.1/14.2	55.9/0	92.7/0
SF	73.7/16.7	94.6/2.4	71.6/0.9	20.0/0
FA	70.1/8.9	86.7/6.7	64.1/0	19.6/0

**Table 3:** Precipitation yield of Pt, Pd, Ru and Rh from three- and four-component model solutions using various reducers: AA, NaBH_4_, SF and FA (the ratio of PGM:PVP:reducer was 1:1:1).

Reducer	P, %			
	Three-Component Solution			
	**Pt**	**Pd**	**Ru**	**Rh**
AA	93.0	40.6	-	68.6
NaBH_4_	97.1	91.5	-	79.9
SF	-	-	-	-
FA	73.7	92.7	-	83.7
	**Four-Component Solution**			
	**Pt**	**Pd**	**Ru**	**Rh**
AA	72.9	54.7	86.9	14.2
NaBH_4_	79.9	94.3	83.0	87.8
SF	57.5	86.4	88.8	35.0
FA	57.6	78.2	83.3	34.1

**Table 4:** Precipitation yield of PGMs and Fe ions from real solutions using NaBH_4_ (ratio of PGM:PVP:NaBH_4_ was 1:1:2 or 1:1:10 for R2 solution).

Real Solution	P, %		
	Pt	Pd	Fe
R1	98.8	-	98.8
R2	100	100	65.2
R3	97.6	100	96.8

**Figure A3:**
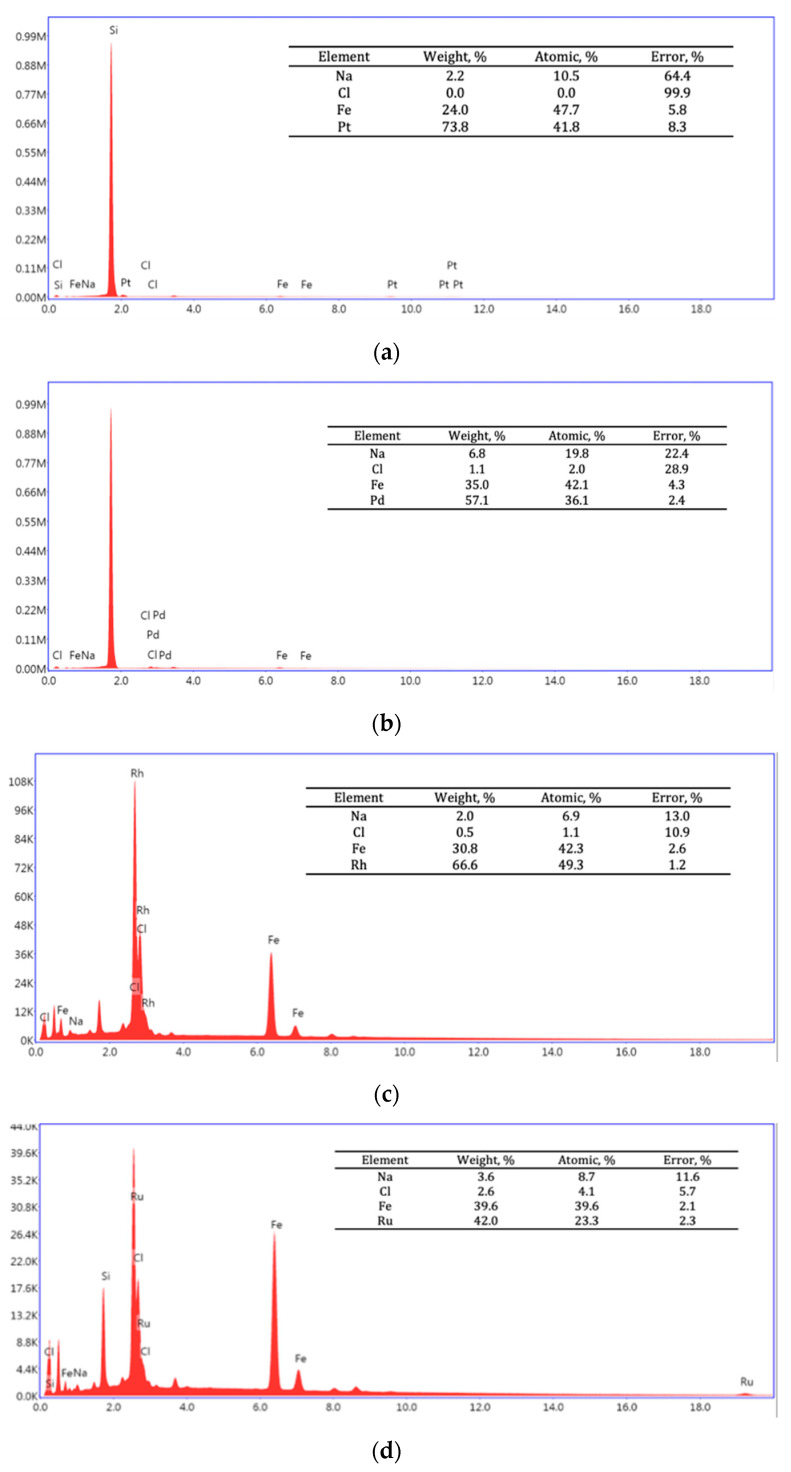
SEM-EDS spectra of (**a**) Pt/Fe NPs, (**b**) Pd/Fe NPs, (**c**) Rh/Fe NPs and (**d**) Ru/Fe NPs from the model solution (the molar ratio of PGM:PVP:NaBH_4_ was 1:1:1).

## Data Availability

The data presented in this study are available on request from the corresponding author.

## Sample Availability

Available from the corresponding authors.

## Appendix A

SEM-EDS images of Pt/Fe NPs after reduction with AA, FA or SF from the model solution are presented in Figure A1.

SEM-EDS images of Pd/Fe NPs, Rh/Fe NPs and Ru/Fe NPs after precipitation with NaBH_4_ from model solution are presented in Figure A2.

SEM-EDS spectra of Pt/Fe NPs, Pd/Fe NPs, Rh/Fe NPs and Ru/Fe NPs after precipitation with NaBH_4_ from the model solution are presented in Figure A3.

Concentrations of metal ions in the feed solutions are shown in Table A1.

**Table A1:** Metal ion concentrations in the real solutions used as feeds for the precipitation of metal nanoparticles.

Chemical Element	Concentration of Metal Ions, mg/dm^3^		
	R1	R2	R3
Pt(IV)	247.0	19.0	89.4
Pd(II)	-	80.0	67.6
Rh(III)	0	0	0
Fe ions	30.1	61.0	80.0
Mg(II)	0.64	0.5	1.0
Zn(II)	6.9	0.2	4.3
Cu(II)	0.6	0.9	0.9

The conversion of NPh at pH 11 and 14 depending on the type of the non-precious metals (Fe, Cu, and Zn) is presented in Table A2.

**Table A2:** Conversion of NPh at pH 11 and 14 depending on the type of the non-precious metals (Fe, Cu, and Zn). Catalyst mass: 1 mg.

Reducer	Conversions of NPh, % at pH 11/14			
	Fe	Cu	Mg	Zn
AA	-	56.6/8.4	-	0.0/0.8
NaBH_4_	5/7.7	93.9/9.2	-	0.5/3.0
SF	3.3/1.6	46.8/0.0	-	0.0/15.6
FA	12.3/12.0	87.5/0.0	-	0.0/3.0

The conversion of NPh at pH 11 and 14 depending on the type of PGM NPs and the addition of a non-precious metal, Fe, Cu, or Zn, is presented in Table A3.

**Table A3:** Conversion of NPh at pH 11 and 14 depending on the type of PGM NPs and the addition of a non-precious metal: Fe. Catalyst mass: 1 mg.

Amount of Catalyst, mg	Conversions of NPh, % at pH 11/14			
	Pt/Fe	Pd/Fe	Ru/Fe	Rh/Fe
1	63.8/95.5	100/100	-	100/96.6
2	100/95.5	100/96.4	-	100/95.0
3	100/95.8	100/96.2	-	100/96.4

The conversion of NPh at pH 11 and 14 depending on the type of three- or four-component NPs is presented in Table A4.

**Table A4:** Conversion of NPh at pH 11 and 14 depending on the type of three- or four-component NPs.

Reducer	Conversion of NPh, % at pH 11/14
	Pt/Pd/Rh
AA	88.7/88.2
NaBH_4_	92.8/94.2
FA	80.1/79.7
	**Pt/Pd/Rh/Ru**
AA	92.0/91.9
NaBH_4_	78.8/97.4
SF	88.1/69.1
FA	85.5/97.0

The conversion of NPh at pH 11 and 14 using a catalyst, precipitated from real solutions after 30 min, is presented in Table A5.

**Table A5:** Conversion of NPh at pH 11 and 14 using catalyst from real solutions after 30 min.

Amount of Catalyst, mg	Conversions of NPh, % at pH 11/14		
	R1	R2	R3
1	39.3/-	0.0/0.0	13.3/96.7
3	42.2/-	0.0/0.0	86.0/97.6
